# Hypothyroidism among Female Medical Students in a Teaching Hospital: A Descriptive Cross-sectional Study

**DOI:** 10.31729/jnma.7582

**Published:** 2022-07-31

**Authors:** Muna Kadel, Pravakar Dawadi, Sabina Khadka, Gita Kumari Yadav

**Affiliations:** 1 Department of Anatomy, Nepalese Army Institute of Health Sciences, Sanobharyang, Kathmandu, Nepal; 2 Nepalese Army Institute of Health Sciences, Sanobharyang, Kathmandu, Nepal; 3 B. P. Koiraia Institute of Health Sciences, Dharan, Sunsari, Nepal

**Keywords:** *hypothyroidism*, *medical student*, *prevalence*, *thyroid stimulating hormone*, *thyroxine*

## Abstract

**Introduction:**

Hypothyroidism is a common clinical condition of thyroid hormone deficiency and is frequently seen in women. Studies regarding the prevalence of hypothyroidism among healthy young adult females are very less. This study aimed to find out the prevalence of hypothyroidism among female medical students in a teaching hospital.

**Methods:**

A descriptive cross-sectional study was conducted among first to final year female medical students in a teaching hospital from 15 August 2021 to 22 January 2022. Ethical approval was obtained from the Institutional Review Committee (Registration number: 296). A semi-structured questionnaire was filled out by the students followed by a clinical examination to recognize the highrisk group by Zulewski's scoring criteria. Blood samples were taken from those who were having a score of >5 points for the thyroid function test to confirm hypothyroidism. Convenience sampling method was used. Point estimate and 95% Confidence Interval were calculated.

**Results:**

Among 141 female medical students, hypothyroidism was found in 3 (2.12%) (0-4.50, 95% Confidence Interval).

**Conclusions:**

The prevalence of hypothyroidism among the female medical students in a teaching hospital was lesser when compared with other studies from similar settings.

## INTRODUCTION

Hypothyroidism is a broad clinical condition ranging from an asymptomatic state to myxedema, multisystem failure with normal or altered levels of thyroxine (T4) and triiodothyronine (T3) and mildly elevated levels of serum thyroid-stimulating hormone (TSH).^1^ The classical signs and symptoms of hypothyroidism are delayed ankle reflex, dry skin, slow movements, diminished hearing, puffiness around the eyes etc.^2^

Thyroid diseases are the most common endocrine diseases in females of reproductive age.^3^ Hypothyroidism in young women is linked to menstrual irregularities, polycystic ovarian disease, miscarries and even infertility. Early diagnosis and treatment remain the cornerstones of management.^4,5^ Prevalence of hypothyroidism in young Nepalese women are less studied.

This study aimed to find out the prevalence of hypothyroidism among female medical students in a teaching hospital.

## METHODS

A descriptive cross-sectional study was conducted in the Nepalese Army Institute of Health Sciences (NAIHS), College of Medicine, Sanobharyang, Kathmandu, Nepal from 15 August 2021 to 22 January 2022. The ethical approval was taken from the Institutional Review Committee of NAIHS (Registration number: 296). The study was conducted on female medical students of MBBS first to final year after taking written informed consent. Female medical students who were present at the time of the study and who had given voluntary consent were included in the study. Convenience sampling method was used.

The sample size was calculated using the following formula:


$$\begin{array}{ll} \text{n} & ={\text{Z}}^{2}\times \frac{\text{p}\times \text{q}}{{\text{e}}^{\text{2}}}\\ & =1.96^{2}\times \frac{0.10\times 0.90}{0.05^{2}}\\ & =139 \end{array}$$

Where

n = minimum required sample size
Z = 1.96 at 95% Confidence Interval (CI)
p = prevalence of hypothyroidism among medical female students taken as 10% (educated guess)
q = 1-p
e = margin of error, 5%

The minimum calculated sample size is 139. However, we have taken 141 female students. According to Zulewski's scoring criteria, clinical hypothyroidism was screened by filling out the semi-structured questionnaire according to responses given by students regarding demographic variables, medical history, family history, symptoms, menstrual history, etc. followed by the subsequent clinical examination inside a well-lit environment of the classroom.^2^ The score included 7 symptoms and 5 signs. Each symptom and sign was given a score of 1 with a total score of 12. A score of >5 points was considered as clinical hypothyroidism. The blood sample was collected from those who had a score of >5 points for biochemical confirmation.

Thyroid Stimulating Hormone (TSH) estimation was done in SRL diagnostic Lab to confirm hypothyroidism In the laboratory, the blood was allowed to clot and was centrifuged at 4000 rpm for 5 minutes. The centrifuged blood in the gel tube contained serum in the upper portion whereas blood cells in the lower portion. The serum (100-200 μl) was used for the hormone assay immediately after centrifugation. Hormone assay for TSH was done by Chemiluminescence immunoassay (CLIA). CLIA is the chemiluminescent hormone assay with immunological reactions in which a chemical probe was used for the emission of light. Patients with serum free T4 <0.89 ng/dl and TSH >5.50 μU/ml, were categorized as hypothyroid.^1^ Elevated TSH with normal T3 and T4 were classified as subclinical hypothyroidism.

Data were collected and analysed by using IBM SPSS Statistics 20.0. Point estimate and 95% Confidence Interval were calculated.

## RESULTS

Out of 141 female medical students, the prevalence of hypothyroidism was 3 (2.12%) (0-4.50, 95% CI) students. The mean age of the female students having hypothyroidism was 20 ±1 years. Out of them 2 (66.67%) students had subclinical hypothyroidism. One (33.33%) participant had clinical hypothyroidism and was under medication (Table 1).

**Table 1:** Hypothyroidism among female medical students (n= 3).

Type	n (%)
Subclinical hypothyroidism	2 (66.67)
Clinical hypothyroidism	1 (33.33)

In this study, all 3 (100%) girls showed coarse skin, cold skin, constipation, increase in body weight, delayed ankle reflex and paraesthesia.

## DISCUSSION

This study shows that the prevalence of hypothyroidism among female medical students is 2.12%. In this study, two students have subclinical hypothyroidism. This finding is similar with the findings of a study conducted in County Durham, Whickham.^6^ The prevalence of undetected hypothyroidism was 3.47% i.e., almost one-third of the hypothyroid patients (186 out of 587) were diagnosed for the first time during the course of study-related screening.^1^ This finding was similar to the findings of this study. Study from three eastern districts of Nepal also reported that 19.5% and 1.1% of the sample population had subclinical hypothyroidism and overt hypothyroidism respectively.^7^ In the study conducted in Scotland the prevalence of hypothyroidism in the young people less than 22 years of age was 0.135% which was less as compared to this study.^8^ This could be attributed to the enrollment of less younger participants than our study.

Prevalence of subclinical hypothyroidism in Duwakot community hospital was 11.44% which is higher as compared to our study.^9^ This contrast finding might be due to the difference in age group. Although the percentage prevalence of thyroid dysfunction among the total population in our study was found to be 2.12%, other studies reported 10.95% and 20.6% of hypothyroidism among the different age groups.^1,7^ In this study, 11.54% of clinical hypothyroidism had biochemical hypothyroid concordance. This was less when compared with another study in which 47% of biochemical subclinical hypothyroid were diagnosed clinically.^2^ This difference may be because of different sample populations and differences in sensitivities for symptoms of hypothyroidism.

This study is a representative sample of female medical students from only one teaching hospital. Due to small sample size the findings cannot be generalised to the large group of the female population.

## CONCLUSIONS

The prevalence of hypothyroidism among the female medical students of the teaching hospital was lesser when compared with other studies from similar settings. Studies determining age adjusted prevalence in the female population with large sample size is recommended for future studies which could give a better understanding of the condition in this population.
